# A globally occurring indel polymorphism in the promoter of the *IFNA2 *gene is not associated with severity of malaria but with the positivity rate of HCV

**DOI:** 10.1186/1471-2156-9-80

**Published:** 2008-12-03

**Authors:** Cristina Tena-Tomás, Iara de Messias-Reason, Le H Song, Jürgen Tomiuk, Peter G Kemsner, Jürgen FJ Kun

**Affiliations:** 1 Department of Parasitology, Institute for Tropical Medicine, Wilhelmstr. 27, 72074 Tübingen, Germany; 2 Hospital de Clinicas, Federal University of Paraná, Curitiba-PR, Brazil; 3 Tran Hung Dao Hospital, No. 1, Tran Hung Dao Street, Hanoi, Vietnam; 4 Department of Medical Genetics, Division of General Human Genetics, Institute of Human Genetics, Wilhelmstr. 27, 72074 Tübingen, Germany; 5 Medical Research Unit, Albert Schweitzer Hospital, Lambaréné, Gabon

## Abstract

**Background:**

Type I Interferons (IFNs) are well known cytokines which exert antiviral activity, antitumor activity and immunomodulatory effects. Single-nucleotide polymorphisms (SNP) and deletions in the gene coding for *IFNA2 *have been shown to influence the level of expression *in vitro*. The indel polymorphism -305_-300delAACTTT showed the strongest effect *in vitro*. To analyse the worldwide distribution of this polymorphism we analyzed five different populations (586 Vietnamese, 199 Central Africans, 265 Brazilians, 108 Kaingang and 98 Guarani). To investigate a possible association with susceptibility to infectious diseases we determined the polymorphism in malaria patients suffering either mild or severe malaria and in a cohort of hepatitis C virus infected individuals.

**Results:**

We could detect the indel polymorphism in all populations analysed. There was no association with this polymorphism and the outcome of malaria but we found an increase of this indel polymorphism in hepatitis C virus positive individuals compared to healthy controls (p = 0.014).

**Conclusion:**

Polymorphisms in genes involved in the interferon pathway have been implicated in the resistance or susceptibility against cerebral malaria and HBV. Here we show that an indel polymorphism, which mediates a disadvantageous effect in HBV patients, may also play a disadvantageous role in HCV infections stressing the importance of a fully functional interferon pathway.

## Background

Interferons (IFNs) constitute a family of secreted cytokines, which are expressed as an early response to various stimuli [1] in the first line of defense against viral infection [2]. The IFN family was originally recognized for its capacity to protect naïve cells against viral infection and IFN-α is known to play an important role in regulating and linking both the innate and the adaptive arms of immunity [3]. IFNs exhibit a broad diversity of biological functions, as represented by three major biological activities: antiviral activity, antitumor activity and immunomodulatory effects [4,5]. The type I IFN family in humans comprise, in essence, 14 genes [6] coding for 13 interferons, and one IFN-beta and one IFN-omega [7]. They all lack introns and are clustered on the short arm of the chromosome 9 [8]. The IFN genes express different proteins that exhibit remarkably different activity profiles [9] and especially IFN-α2 has an important impact on the clinical field for the treatment of viral infections and different forms of cancer [6].

Recently it was shown that the genes *IFNAR2 *and *IL10RB *involved in binding interferons or interleukin 10-related cytokines, respectively were linked to persistence of hepatitis B virus in The Gambia [10]. This finding gave us a rational to look at other genes involved in the interferon pathway.

It has been shown that there is individual variation in the production of IFN-α [11] which could be due to mutations in the promoter of the *IFNA2 *gene. Song et al. (2006) showed that naturally occurring alterations in the promoter of this gene, especially the deletion -305_-300delAACTTT, reduced the transcription of this gene *in vitro*. This reduction could explain the individual differences in the interferon levels being one of the possible causes of susceptibility of hepatitis B [12].

In this paper we examined the level of genetic differentiation of the promoter of the *IFNA2 *gene by DNA sequencing and compared allele frequencies and genotype distributions in populations from Central Africa (Gabon), Brazil and Vietnam. One of these populations consisted of patients having malaria of different severity (Central Africans) [13]; the other was affected by hepatitis C virus (HCV) (Brazilians of Caucasian descent) [14]. With these patient cohorts we sought to test the hypothesis whether the indel polymorphism in the promoter of the *IFNA2 *gene is associated with disease outcome of other infectious diseases. Healthy Amerindians were included to investigate the frequency of this mutation and to detect a possible factor leading to an increased risk to contract infectious diseases [15].

## Methods

### Patients

1256 individuals were enrolled in this study at different sites: 586 healthy blood donors from the ethnic group of the Kinh at the Than Hung Dao Hospital in Hanoi as an extension of the study described in [12], 199 Central Africans at the Albert Schweitzer Hospital in Lambarènè [13] and 265 Brazilians of Caucasian origins at the Hospital de Clinicas in Curitiba [14], 108 Kaingang and 98 Guarani were recruited in their villages. Kaingang and Guarani are the two major Amerindian tribes living in Southern Brazil but despite living side by side for centuries the two groups differ in many aspects of culture and genetics; e. g. HLA type [16].

The Brazilians of European descent were either patients enrolled in a hepatitis C treatment study (n = 101) or healthy controls (n = 164) [14]. No patients had a history of alcohol or drug abuse. Patients were included into the study when HCV antibodies and HCV RNA was detected in serum; the presence of HBV and HIV antibodies was an exclusion criterion. From all patients biopsies were taken and liver histology was assessed blindly by two pathologists and staging of fibrosis.

Central Africans individuals were affected either by mild (n = 100) or severe malaria (n = 99) [13]. Mild malaria was characterised by a parasitaemia < 50,000 parasites/μl, haemoglobin > 80 g/L, glucose > 50 mg/dL, lactate < 3 mmol/L, leukocytes < 12/nL, platelets > 50/nL, no schizontaemia, and no signs of severe malaria such as cerebral malaria or other infections. The severely infected children presented with severe anaemia or hyperparasitaemia as the main complications of clinical disease. Appropriate treatment with sulfadoxine/pyrimethamine was given to mild cases. Severe cases were treated with intravenous quinine plus clindamycin.

Amerindians were healthy subjects from a human genetics study [17].

The genetic analysis was approved by the relevant ethic committees of the involved institutions: the Institutional Review Board of the Tran Hung Dao Hospital, Hanoi, Vietnam; the ethics committee of the Medical Faculty in Tübingen, Germany, the ethics committee of the Hospital de Clínicas in Curitiba, Brazil and the ethics committees of the International Foundation for the Albert Schweitzer Hospital in Lambaréné, Gabon.

### DNA extraction

Blood from all the participants was collected in EDTA tubes and plasma was separated, aliquoted and stored at -20°C for later use. Genomic DNA was extracted from blood samples from using the QIAamp DNA blood kit (Qiagen, Hilden, Germany), as described in the user manual.

### Polymerase chain reaction (PCR)

Genomic DNA was used as template for PCR. A single fragment of 480 bp from the *IFNA2 *promoter was amplified using the following primer sets: IFN-F: 5'-TTTCAAAAAAGTTGCTCTAAG-3', IFN-R: 5'-GTAGATGTTGCAGATGCTGCT-3' (Operon, Germany).

All amplifications were initiated with a denaturing step of 94°C for 5 min, followed by 42 cycles of 94°C for 30 sec, 45°C for 30 sec annealing time and 72°C for 1 minute elongation. The reaction was stopped after a final elongation step of 72°C for 10 min. A PTC-200 Peltier Termal Cycler (Bio-Rad, Munich, Germany) was used to amplify all the samples.

### Genotyping

After purification, the respective PCR products were sequenced with the same primers described above using BigDye terminator chemistry on an ABI PRISM 3100 Genetic analyzer (Applied Biosystems, Foster City, USA). The sequences were analyzed for polymorphisms in the promoter of the *IFNA2 *gene after DNA alignment of the sequenced products using the Bioedit alignment program (North Carolina State University, USA). The quality of the sequencing was assessed by visual inspection with reference to the electropherograms.

### Statistical analysis

*IFNA2 *allele frequencies and genotype distributions for each population as well as Hardy-Weinberg exact test were calculated using the Genepop version 3.4 .

Differences in allele frequencies and genotype distributions between populations were calculated using Fisher's exact test using STATA . *P*-value < 0.05 was considered to be significant.

## Results

### Allele frequency

Of the four mutations in the *IFNA2 *promoter only the deletion -305_-300delAACTTT was analyzed because it was shown to influence significantly the promoter activity *in vitro *[12]. The frequency of this deletion in Central Africans, Brazilians, Guarani, Kaingang and Vietnamese is presented in Table 1. Overall, 2512 alleles were analyzed, and 2246 out of 2512 (90%) had no deletion while 266 (10%) of the alleles carried the deletion.

**Table 1:** Number of alleles and frequency of the indel polymorphism in the *IFNA2 *promoter in five populations.

	Major allele	del -301/-306	Total
Central Africans	376 (0.94)	22 (0.06)	398
Brazilians	447 (0.84)	83 (0.16)	530
Guarani	173 (0.88)	23 (0.12)	196
Kaingang	202 (0.93)	14 (0.07)	216
Vietnamese	1048 (0.89)	124 (0.11)	1172

Total	2246 (0.90)	266 (0.10)	2512

Data are given in numbers of chromosomes analysed and frequencies in brackets.

del -301/-306 indicates the polymorphism -305_-300delAACTTT.

Allele frequencies at this locus were compared for the five groups showing an irregular distribution in the different populations (*P *< 0.001). Therefore pairwise comparison of the allele frequencies was performed as shown in Table 2. The frequency of the deletion was significantly higher in Brazilians than in any other population except for the Guarani (*P *= 0.208). In Central Africans we find a significantly lower frequency of this variant allele compared with the rest of the populations except for Kaingang (*P *= 0.725).

**Table 2:** Pairwise comparison of the allele frequency of the indel polymorphism in the *IFNA2 *promoter.

POPULATION	Brazilians	Guarani	Kaigang	Vietnamese	Central Africans
Brazilians	--	--	--	--	--
Guarani	0.208	--	--	--	--
Kaingang	< 0.001	0.080	--	--	--
Vietnamese	0.004	0.609	0.072	--	--
Central Africans	< 0.001	0.013	0.725	0.004	--

Given are *P*-values obtained by Fisher's test.

### Genotype distribution

The results of the observed genotype distribution in the five populations are shown in Table 3. Among the 1256 individuals enrolled in the study, 1010 (80.4%) had no deletion, 226 (18%) were heterozygous and 20 (1.6%) carried the deletion on both chromosomes (Table 3). The highest proportion of homozygous individuals carrying the deletion was found in the Brazilian population (5%). In contrast no homozygous individuals were found in the Guarani being the population with the highest number of heterozygous individuals. We performed pairwise comparison of the genotypes frequencies among the five groups (Table 4). Brazilians and Guarani did not significantly differ in genotype distribution whereas Central Africans were significantly different from any of the populations except for the Kaingang.

**Table 3:** Genotypic distribution of the indel polymorphism in the *IFNA2 *promoter in five populations.

	wt/wt	wt/del -301/-306	del -301/-306/del -301/-306	Total
Central Africans	178 (89.5)	20 (10)	1 (0.5)	199 (100)
Brazilians	195 (73.5)	57 (21.5)	13 (5)	265 (100)
Guarani	75 (76.5)	23 (23.5)	0 (0)	98 (100)
Kaingang	96 (88.9)	10 (9.3)	2 (1.8)	108 (100)
Vietnamese	466 (79.5)	116 (19.8)	4 (0.7)	586 (100)

Total	1010 (80.4)	226 (18)	20 (1.6)	1256 (100)

Data are given in number with percentages in brackets. del -301/-306 indicates the polymorphism -305_-300delAACTTT. wt indicates the major allele.

**Table 4:** Pairwise comparison of the genotypic distribution of the indel polymorphism in the *IFNA2 *promoter.

POPULATION	Brazilians	Guarani	Kaingang	Vietnamese	Central Africans
Brazilians	--	--	--	--	--
Guarani	0.215	--	--	--	--
Kaingang	0.002	0.091	--	--	--
Vietnamese	0.004	0.703	0.054	--	--
Central Africans	< 0.001	0.010	0.736	0.002	--

Given are *P*-values obtained by Fisher's test

### Hardy-Weinberg equilibrium

Expected genotype distributions were calculated using GenePop for the position which was polymorphic in the study populations (data not shown). The expected numbers did not significantly differ from the observed numbers in Central Africans, Guarani and Vietnamese, suggesting that these populations were in Hardy-Weinberg equilibrium. In contrast, exact tests indicated that expected and observed genotype distributions did significantly differ in Brazilians and Kaingang, indicating that these two groups are indeed in Hardy-Weinberg disequilibrium (Table 5).

**Table 5:** Deviation from Hardy-Weinberg equilibrium in the five populations.

Population	p-value
Central Africans	0.460
Brazilians	0.004
Guarani	0.350
Kaingang	0.004
Vietnamese	0.274

Given are *P*-values obtained by Fisher's test

### Association of the deletion Δ-300/-305 with clinical aspects

To determine the association of the deletion -305_-300delAACTTT with different infectious diseases, we compared the frequency of *IFNA2 *with clinical manifestation of the diseases as well as with treatment outcome. No significant association of the mutation with severity of malaria (mild versus severe) were found in the Central African populations (allelic frequencies of the deletion in mild malaria cases 0.10 (90 individuals without deletion, 9 with the deletion on one chromosome) versus 0.12 in severe cases (89 individuals without deletion, 9 with the deletion on one chromosome, 1 individual with the deletion on both chromosomes, P = 1). There was a statistically significant association with the manifestation of HCV in Brazilians of Caucasian descent. We found among 101 HCV positive individuals 68 (67%) without deletion on any chromosome; 30 (30%) with a deletion on one chromosome and 3 (3%) with a deletion on both chromosomes. In the healthy control group we had in a total of 265 individuals 127 (77%) with no deletion on any chromosome; 27 (17%) with a deletion on one chromosome and 10 (6%) with a deletion on both chromosomes. The difference in the distribution is statistically significant (p = 0.03). When looking at the heterozygous individuals we find an odds ratio of 2 (CI = 1–4; p = 0.014) when we compare patients with healthy controls. The deletion was not found to be significantly associated with the treatment success of IFN-α with or without ribavirin in hepatitis C patients (data not shown).

## Discussion

In this study we analyse a widely distributed chromosomal deletion in the promoter of the *IFNA2 *gene in five cohorts. It has been shown that the deletion -305_-300delAACTTT is associated with the clinical presentation of hepatitis B and a lower expression level *in vitro *[12]. Here we investigated a possible influence of this deletion in malaria and hepatitis C. There is evidence that IFN-α can induce NOS2 expression and NO production by human mononuclear cells *in vitro *and *in viv*o [18]. Also NO has been reported to have antiviral activity for a variety of viruses [19] and to play an important role in malaria [13,20]. Vigário *et al*. have shown that recombinant human IFN-α inhibits cerebral malaria and reduces parasite burden in mice [21].

Additionally, IFN-α, either alone or in combination with ribavirin, is currently the standard treatment of patients with hepatitis C [22]. Therefore we speculated that the individual level of IFN-α depending on the promoter activity of the gene might affect the outcome of malaria or the therapy success in hepatitis C. In the latter event the intrinsic production would have supported the externally given IFN-α. We could not detect an influence of this polymorphism in Central Africans children with malaria. To detect a statistically significant distribution with frequencies in the malaria cohorts of 0.12 and 0.10 much larger cohorts would be needed.

Treatment success of HCV infection was not influenced by the presence of the polymorphism. We could, however, detect a difference in distribution of genotypes in HCV positive and healthy individuals. This finding confirms an earlier observation in hepatitis B patients stressing the importance of *IFNA2 *expression in these diseases.

The populations studied here diverged a long time ago and have undergone diverse selective pressure, explaining the genetic differences or similarities between groups. This indel polymorphism probably has an ancient origin and has entered the American continent 30.000 years ago [23] with the migration wave from Asia. Vietnamese and the two Amerindian groups, Kaingang and Guarani, therefore do not differ significantly in the genotype distribution. The Kaingang, unlike all other groups, do not show a significantly different genotype distribution to the Central Africans population, furthermore the Kaingang also diverge from the Hardy-Weinberg equilibrium. This finding is partly due to the traffic of slaves to South America during the 16^th ^to the 19^th ^century and a subsequent absorption of escaped African slaves by the Kaingang. By comparing blood groups and protein polymorphisms it was shown that Kaingang population had a higher proportion of non-Amerindian alleles than the Guaranis [24]. We found also a violation of the Hardy-Weinberg equilibrium in Brazilian, which is probably due to admixture in this area caused by the migration of individuals from different parts of Europe [25].

## Conclusion

The polymorphism analysed in this work influences the course of hepatitis B and the prevalence of HCV. The treatment success in hepatitis C was not influenced by this indel polymorphism. Obviously, the externally given IFNα concentration is much higher than the intrinsic production of the patients. However, the fact that the indel polymorphism has is more frequently present in hepatitis B and C viruses argues for the importance of the IFNα pathway in both viral infections.

Human malaria was not influenced by the indel polymorphism; IFNα probably plays a role at the beginning of an infection but not at later time points when the parasite has manifested itself.

## Authors' contributions

CTT carried out the molecular genetic experiments, participated in the sequence alignment and drafted the manuscript. IMR supervised the HCV study. LHS participated in the sequence analysis. JT was involved in population genetics analysis. PGK designed the clinical study on malaria and supervised the performance. JFJK supervised the molecular work, was involved in the statistical analysis and finalized the manuscript.

All authors read and approved the final manuscript.
